# Quantitative Trait Loci Mapping and Comparative Transcriptome Analysis of Fruit Weight (FW) in Watermelon (*Citrullus lanatus* L.)

**DOI:** 10.3390/genes15070933

**Published:** 2024-07-17

**Authors:** Song Guo, Mei Tian, Huiying Du, Shengfeng Liu, Rong Yu, Huolin Shen

**Affiliations:** 1 Horticulture College, China Agricultural University, Beijing 100193, China; 2001929@163.com; 2 Institute of Horticultural Research, Ningxia Academy of Agriculture and Forestry Sciences, Yinchuan 750002, China; tmei-2002@163.com (M.T.); du.huiying2008@163.com (H.D.); shengfeng.liu@163.com (S.L.)

**Keywords:** watermelon, fruit weight, genetic map, QTL mapping, bulked segregant analysis (BSA), RNA-seq, differential expression analysis

## Abstract

The watermelon (*Citrullus lanatus* L.) holds substantial economic value as a globally cultivated horticultural crop. However, the genetic architecture of watermelon fruit weight (FW) remains poorly understood. In this study, we used sh14-11 with small fruit and N14 with big fruit to construct 100 recombinant inbred lines (RILs). Based on whole-genome resequencing (WGR), 218,127 single nucleotide polymorphisms (SNPs) were detected to construct a high-quality genetic map. After quantitative trait loci (QTL) mapping, a candidate interval of 31–38 Mb on chromosome 2 was identified for FW. Simultaneously, the bulked segregant analysis (BSA) in the F2 population corroborated the identification of the same interval, encompassing the homologous gene linked to the known FW-related gene *fas*. Additionally, RNA-seq was carried out across 11 tissues from sh14-11 and N14, revealing expression profiles that identified 1695 new genes and corrected the annotation of 2941 genes. Subsequent differential expression analysis unveiled 8969 differentially expressed genes (DEGs), with 354 of these genes exhibiting significant differences across four key developmental stages. The integration of QTL mapping and differential expression analysis facilitated the identification of 14 FW-related genes, including annotated TGA and NAC transcription factors implicated in fruit development. This combined approach offers valuable insights into the genetic basis of FW, providing crucial resources for enhancing watermelon cultivation.

## 1. Introduction

The watermelon (*Citrullus lanatus* L.) (2*n* = 2x = 22) stands as a vital cash crop globally, renowned not only for its widespread cultivation but also as a favored fresh fruit replete with health-enhancing compounds such as vitamin C, lycopene, and β-carotene [1]. The annual worldwide production of watermelon surpasses 100 million tons, solidifying its significance in the agricultural landscape. Notably, China emerges as the leading producer and consumer of watermelon, boasting an extensive harvested area exceeding 1.47 million hectares and a production exceeding 60 million tons in 2019. Simultaneously, the watermelon production in China reached 63.241 million tons, accounting for 60.61% of the global total.

High-density genetic maps serve as invaluable tools for quantitative trait loci (QTL) mapping and candidate gene identification. In the early stages, genetic linkage maps for watermelon relied on various markers, such as isozymes [2,3], amplified fragment length polymorphisms (AFLPs), and simple sequence repeat (SSR) [4,5]. With advanced sequencing technology and decreased costs, next-generation sequencing (NGS) has facilitated the application of single nucleotide polymorphism (SNP) markers [6]. BSA stands out as a time- and cost-efficient QTL mapping approach [7,8]. Various algorithms for BSA have been documented and widely implemented, such as G’ [9], Δ(SNP-index) [10], ED^4^ [11], QTG-seq [12] and DeepBSA [13]. Meanwhile, a high-quality reference genome could greatly enhance the accuracy of QTL mapping. Several reference genomes of watermelon have been reported [14,15,16,17], including a telomere-to-telomere (T2T) gap-free genome [18]. Based on the NGS technology, BSA algorithms and the high-quality genome, several genetic maps were constructed and QTLs were identified in watermelon [19,20,21].

Fruit shape (FS) constitutes an agriculturally important fruit yield trait, and various related QTLs and genes have been documented in cucurbits [22]. As FW is a useful indicator, complementary to fruit length (FL) and fruit width (FD), for describing three-dimensional fruit growth, FW is an important fruit trait worth studying [23]. By constructing segregating populations derived from a cross between WI7238 (long fruit) and WI7239 (round fruit) for QTL mapping, two QTLs, *FS1.2* and *FS2.1*, were found to control FS. These two loci encode the tomato homologs *SUN* (*CsSUN25-26-27a*) [24] and *SlTRM5* (*CsTRM5*) [25], respectively. In watermelon, Sandlin identified 10 QTLs linked to FL, explaining phenotypic variation (PV) ranging from 7.3% to 40.8%. Additionally, eight QTLs associated with FD (ranging from 8.7% to 50.0%) and five QTLs related to fruit shape indices (FSIs) (ranging from 2.8% to 56.6%) were discovered [26]. Subsequent efforts aimed at refining the genetic map through the development of four RILs continued to uncover additional QTLs associated with watermelon FS [27]. Another study identified four QTLs related to the watermelon FSI, elucidating 31.9% of the PV, thus contributing to a more comprehensive understanding of the genetic underpinnings of watermelon FS [28]. In several recent studies, *ClSUN25-26-27a* was identified as a putative candidate gene co-localizing with *ClFSI3.1* [29,30].

Although many QTLs related to FW have been found in watermelon, few key genes have been confirmed. Therefore, combining QTL mapping and differential gene expression analysis is a potential way to mine FW key genes. In this study, the construction of 100 RILs from sh14-11 and N14, coupled with QTL mapping, led to the identification of the chromosome 2 interval (31–38 Mb) as a candidate region influencing FW in watermelon. Notably, consistent results were obtained through BSA in the F_2_ population, further corroborating the significance of this interval. Subsequent differential expression analysis across eleven crucial developmental stages between sh14-11 and N14 unveiled a total of 8969 DEGs, with 354 genes exhibiting variations in the key stages. Finally, 14 genes within the QTLs were mined for FW and annotated. We believe this study is of great significance for the genetic understanding and cultivation improvement of FW in watermelon.

## 2. Materials and Methods

### 2.1. Plant Material and DNA Extraction

The female line (N14) and the male line (sh14-11) are cultivated inbred lines of watermelon from the Institute of Horticulture Research, Ningxia Academy of Agricultural and Forestry Sciences (NAAFS), whose fruit weight is stable. The mapping populations were generated from a cross between N14 and sh14-11, in which the F2 segregation population consisted of 100 individual plants that were planted in NAAFS in the spring of 2021. The recombinant inbred lines (RILs, F5), consisting of 2 parents and 100 offspring, were derived by single seed descent and were planted in NAAFS during the winter of 2022. In the watermelon field, all plants were grown under identical conditions. The soil was uniformly prepared and fertilized, and a consistent watering schedule was maintained. The plants were spaced evenly to avoid competition. The pest control measures, light exposure and temperature were standardized. This ensured that any observed differences in fruit size were due to genetic or experimental variables rather than environmental factors. Each fruit from the plant at the mature stage was weighed using an electronic scale, with each sampling repeated 5 times. The weight of each fruit at the mature stage was recorded to two decimal places.

Leaf tissue from both the F_2_ population and the RIL population was extracted using the CTAB method, with slight modifications. Twenty individual lines with significantly large fruits and another 20 lines with notably small fruits from the F_2_ population were chosen based on the subsequent bulked segregant analysis (BSA). The DNA extracted from both parents and 100 RILs was utilized in the construction of the genetic map.

### 2.2. High-Throughput Sequencing and Data Processing and Analysis

The DNA libraries underwent terminal repair for sequencing, including the addition of a 3′ A and a sequencing linker. Subsequently, the samples were purified and amplified via PCR. Paired-end sequencing was conducted using the HiSeq2500 system (Illumina, San Diego, CA, USA). Raw paired-end sequencing reads were initially preprocessed to eliminate reads with adapters and excessively low-quality pair reads (q < 10). Then, the clean reads were mapped to the *Citrullus_lanatus*. The WCG_v1 watermelon reference genome (http://cucurbitgenomics.org/ftp/genome/watermelon/WCG/v1/ accessed on 14 July 2024) was mapped using the BWA (Burrows–Wheeler Aligner program).

### 2.3. SNP/InDel Identification and Annotation

GATK was utilized for base recalibration, variant calling and for stringent filtering of variants to obtain the final variant clusters. The default parameters were used for variant calling and mapping for all samples. The MarkDuplicates tool was applied to remove duplicated reads originating from PCR amplification. Then, SnpEff was employed to annotate and predict the variation (SNP, Small InDel).

### 2.4. Construction of Genetic Map and Mapping QTL

After identifying the highly qualified SNP/InDel markers, we used HighMap (http://highmap.biomarker.com.cn/ accessed on 14 July 2024) to construct the genetic map, including the steps of linkage grouping, marker sequencing, genotyping error correction and mapping evaluation. During chromosome scanning and genotyping in each window, a 1-SNP step and a 15-SNP sliding window were adopted. The SNPs located between adjacent recombination breakpoints aggregate into recombination bins. These binding markers are helpful to construct linkage groups using HighMap tools.

QTL IciMapping (Version 4.0) software was employed to detect QTLs with genetic maps. The F_2_ individuals displaying band patterns resembling those of the female and male parents were designated as A and B, respectively, while the patterns resembling the F_1_ type were labeled as H. The results were imported into QTL IciMapping for analysis, with the parameter “mapping steps = 1 cM”. The statistical analysis used for the identification of the QTLs included composite interval mapping (ICIM) with a filtering threshold of locus at “LOD > 2.5”.

### 2.5. BSA and Mapping Strategy

Twenty F_2_ individuals with extreme big fruit were carefully chosen to represent the big-fruit pool and twenty F_2_ individuals with extreme small fruit represent the small-fruit pool. Combined with two parent lines, four samples were created to extract DNA and conduct genomic sequencing. The variant sites were called by aligning sequence reads to the *Citrullus_lanatus*.WCG_v1 genome. SNP-index values were calculated for all SNPs across the genome, and QTLs were detected based on these calculations.

### 2.6. Transcriptome Analysis and Candidate Gene Prediction

The RNA-seq of watermelon contained 22 tissue samples, including flowers collected two days before flowering (nF2, shF2), one day before flowering (nF1, shF1) and on the day of flowering (nF0, shF0), as well as 7 days after fruit setting (shR7, nR7, shP7, nP7), 15 days (shR15, nR15, shP15, nP15), 23 days (shR23, nR23, shP23, nP23) and 31 days (shR31, nR31, shP31, nP31). Each tissue underwent three biological replicates, totaling 66 tissue samples. Following library construction, an initial quantification was conducted using the Qubit 2.0 fluorometer, with subsequent dilution of the library to 1.5 ng/μL. The insert size of the library was assessed using an Agilent 2100 bioanalyzer. Upon confirmation that the insert size met the anticipated specifications, accurate quantification of the library’s effective concentration (ensuring it exceeded 2 nM) was carried out through qRT-PCR. This meticulous process was implemented to guarantee the library’s quality before proceeding with Illumina sequencing for the various libraries.

After sequencing, the reads with adapters were removed. Hisat2 was employed to align the reads with the *Citrullus_lanatus* (WCG_v1) genome [17] to obtain the location of the genes and transcripts. Differentially expressed genes (DEGs) among sample groups were identified with DESeq2. To ensure the precision of DEG categorization, we established stringent criteria, with a false discovery rate (FDR) threshold set at ≤0.05 and an absolute log2(ratio) value of ≥1. These thresholds were employed to discern significant differences in gene expression between N14 and sh14-11.

## 3. Results

### 3.1. Population Establishment and Genetic Map Construction

**Figure 1:**
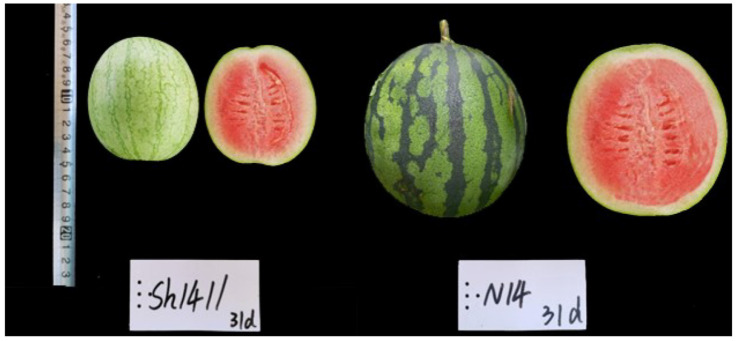
The FW of sh14-11 and N14 on 31 days after fruit setting.

To construct a stable watermelon population exhibiting variable FW, the male parent N14, characterized by large fruit, was crossed with the female parent sh14-11, known for its small FW. The fruit of sh14-11 was about 0.30 kg, while the fruit of N14 was 1.51 kg (Figure 1). Following single seed descent, a population of 100 recombinant inbred lines (RILs) was developed, encompassing a range of fruit from 0.11 kg to 3.84 kg, with each line demonstrating consistent FW.

Following whole-genome resequencing, we procured a total of 10.87 Gb and 10.71 Gb of high-quality reads from the respective parental inbred lines. The RIL population, comprising 100 individuals, contributed to a cumulative 219.68 Gb of high-quality reads. The markers displayed an average coverage depth of 23-fold for the male parent, 22-fold for the female parent and 4.23-fold for their offspring (Supplementary Table S1). Remarkably, over 98% of clean reads were effectively mapped to the reference genome. With SNP filtering, a total of 218,127 SNPs were identified, with a minimum 4-fold depth in the parent lines. This dataset was employed to construct a high-density genetic map consisting of 11 linkage groups (LGs) (Table 1).

### 3.2. Evaluating the Genetic Map and Mapping QTL for FW

**Figure 2:**
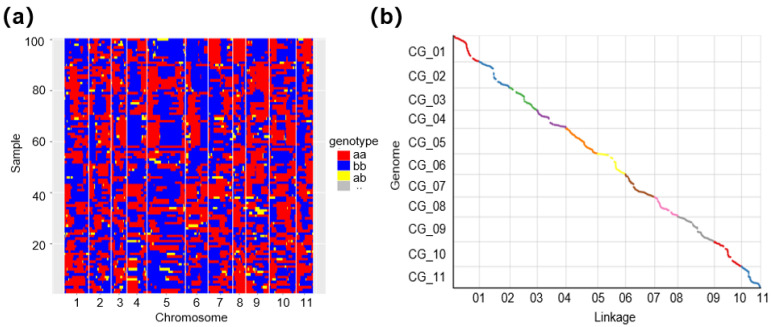
Haplotype map and genetic map evaluation of RILs. (**a**) The haplotype map of RILs; aa and bb stand for homozygosity, and ab stands for heterozygosity. (**b**) The collinearity between genetic map and physical map.

Individual haplotype maps were created for 100 RILs, successfully outlining nearly all recombination blocks (Figure 2a). To assess the genetic map’s quality, we examined the collinearity between the genetic map and the physical map across the 11 chromosomes. It is noteworthy that all Spearman correlation coefficients for linkage groups (LGs) exceeded 0.995, signifying a high degree of collinearity between the genetic map and the physical map (Figure 2b).

**Figure 3:**
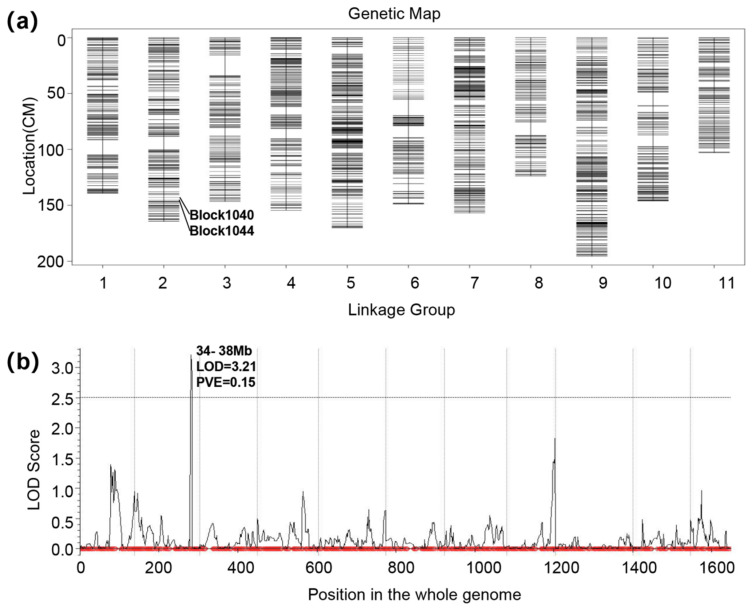
High-density genetic map and quantitative trait loci mapping of FW. (**a**) The high-density genetic map for watermelon. (**b**) Mapping QTL for FW.

The completed high-density genetic map was established across 11 chromosomes, integrating 2597 recombination bins housing 218,127 SNPs and demonstrating a well-dispersed linkage distance (Figure 3a). The cumulative map length reached 1649.42 cM, showcasing an average distance of 0.64 cM between neighboring bin markers. On the whole, the bin markers exhibited a balanced distribution throughout the genome, with nearly 96.19% of intervals between adjacent markers measuring less than 5 cM. LG9 emerged as the most extensive linkage group, covering 195.71 cM and featuring 247 bin markers, with an average inter-marker distance of 0.8 cM. Conversely, LG11 represented the smallest linkage group, covering 102.93 cM and hosting 175 bin markers, with an average distance of 0.59 cM between adjacent markers (Table 1).

The composite interval mapping (CIM) method was employed to pinpoint loci significantly linked to FW. A distinct locus emerged on chromosome 2, reaching maximum LOD scores of 3.21, exceeding the threshold of 2.5 (Figure 3b). This noteworthy locus explained 15.135% of the phenotypic variation in FW, showing an additive effect of 0.246. This locus is located at 143 cM/34 Mb on chromosome 2, which is between block1040 and block1044 from the marker of the genetic map.

### 3.3. Bulked Segregant Analysis for FW

After crossing the parental lines to create the F_2_ population, the FW of all individuals was assessed. The 20 individuals with the largest fruit weight were selected as high pools, and the 20 individuals with the smallest fruit weight were selected as low pools. High-quality sequencing generated a total of 38.78 Gb reads, with an approximate sequencing depth of 23× for each pool. The average Q30 ratio was an impressive 94.9%, and over 98.7% of clean reads were successfully aligned to the reference genome. Following the quality-check procedures, the clean short reads underwent alignment to the reference genome using BWA. Subsequently, employing stringent selection criteria in the QTL-seq pipeline, a total of 384,411 SNPs were identified across all 11 chromosomes.

**Figure 4:**
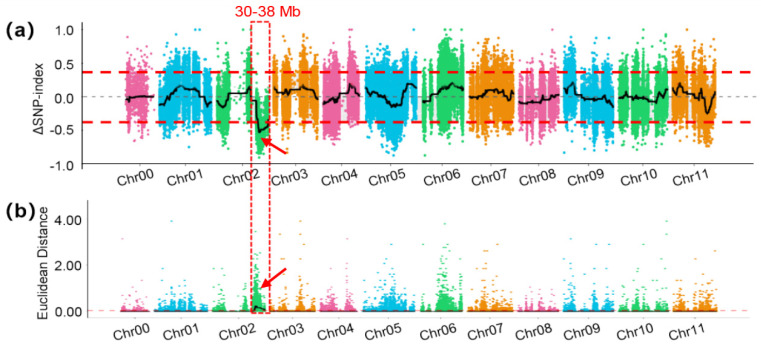
BSA mapping of FW in watermelon. (**a**) Distribution of Δ(SNP-index) across the genome. (**b**) Distribution of Euclidean distance (ED) across the genome. The red dotted box and arrow represent QTL identified by the two methods.

Subsequently, we computed the Δ(SNP-index) value for each SNP and plotted it on the genome. Applying a threshold of 0.4, the locus influencing FW in watermelon was identified (Figure 4a). This locus spans from 30,966,186 bp to 38,769,378 bp on chromosome 2, covering a total of 7.8 Mb. Concurrently, the Euclidean distance (ED) value was calculated and depicted, corroborating the identical genomic region (Figure 4b) and affirming the reliability of the results. Remarkably, the locus identified by BSA in the F_2_ population aligned with the locus identified through map-based cloning in the RIL populations, emphasizing its pivotal role as a significant interval for FW in watermelon.

### 3.4. Multi-Tissue Gene Expression Profiles of Two Parental Lines

To delineate the expression profiles between the parental lines sh14-11 and N14, we collected 22 tissue samples for RNA sequencing. These samples included flowers collected two days before flowering (nF2, shF2), one day before flowering (nF1, shF1) and on the day of flowering (nF0, shF0), as well as fruit pulp and fruit peel collected 7 days after fruit setting (shR7, nR7, shP7, nP7), 15 days after fruit setting (shR15, nR15, shP15, nP15), 23 days after fruit setting (shR23, nR23, shP23, nP23) and 31 days after fruit setting (shR31, nR31, shP31, nP31). Each tissue underwent three biological replicates, totaling 66 tissue samples. After conducting RNA-seq analysis, a cumulative 423.37 Gb of clean data were obtained. The average clean data for each sample reached 5.75 Gb, with the Q30 base percentage being 92.25% or higher. The clean reads from each sample were juxtaposed with the designated reference genome, showcasing a comparison efficiency ranging from 96.48% to 97.87% (Supplementary Table S2).

**Figure 5:**
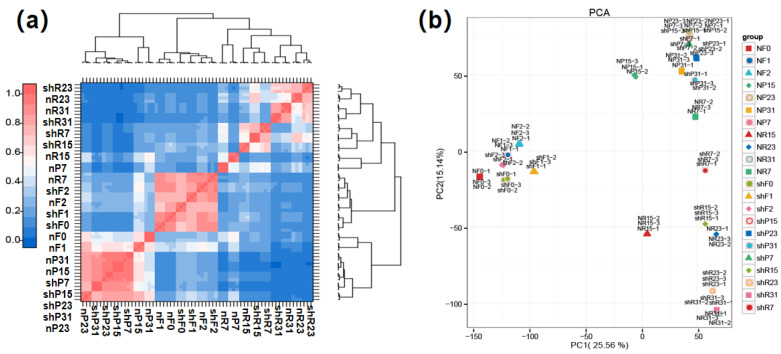
Comparative analysis of three biological repeats in 22 tissues. (**a**) Correlation coefficient between biological repetitions in 22 tissues. (**b**) Principal component analysis clustering performed among 22 tissues.

To assess the consistency among the biological replicates, we employed Pearson’s correlation coefficient as the evaluation index. The findings revealed correlation coefficients between biological replicates of the 22 tissue samples ranging from 0.97 to 1 (Figure 5a, Supplementary Table S3), signifying a high degree of consistency and data reliability among the biological replicates. Simultaneously, a principal component analysis (PCA) was conducted, illustrating that PC1 could explain 25.56% of the variation, and PC2 could explain 15.14% of the variation. Meanwhile, three replicates of the same tissue nearly overlapped, and analogous tissues clustered together, indicating that our data are both in line with the expectations and reliable (Figure 5b).

The structural annotation of genes and transcripts in reference genome is often incomplete, as transcriptome data usually lack annotation. After the identification of continuous mapped reads beyond the initial gene boundaries, prompting the extension of the untranslated region (UTR) both upstream and downstream to rectify the gene boundary, we succeed in optimizing the structure annotation of 2941 genes. Simultaneously, StringTie2 software was employed to splice the mapped reads and compare the results with the original genome annotation. This facilitated the identification of original annotation regions and the exploration of new transcripts and genes within this species. After filtering out sequences with excessively short, coded peptide chains (less than 50 amino acid residues) or those containing only a single exon, 1965 new genes were discovered. Consequently, the expression profile analysis not only rectified inaccuracies in the annotation of previous transcripts but also unveiled new genes. This represents a valuable resource for enhancing our understanding of the watermelon genome.

### 3.5. Identification of Differentially Expressed Genes (DEGs) Related to FW

**Figure 6:**
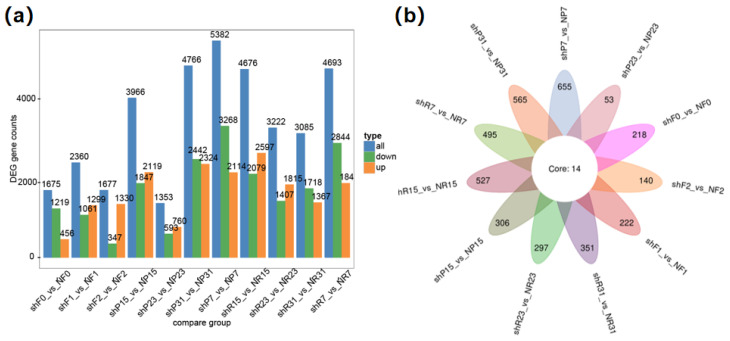
Analysis of differentially expressed genes between sh14-11 and N14 in 11 pairs of tissues, including 3 stages of flower (F0, F1, F2), 4 stages of fruit pulp (R7, R15, R23, R31) and 4 stages of fruit peel (P7, P15, P23, P31). (**a**) Number of differentially expressed genes in 11 corresponding pairs of tissues between parents; (**b**) overlapping differentially expressed genes in 11 corresponding pairs of tissues.

Utilizing high-quality transcriptome data, we identified DEGs in the samples from the corresponding tissues of parental lines. This analysis involved 11 pairs of tissues, revealing a range of 1353 to 5382 DEGs between each pair (Figure 6a). Notably, the fruit peel sampled 7 days after fruit setting exhibited the highest number of DEGs, while the fruit peel sampled 23 days after fruit setting showed the fewest. As FW has changed dramatically during this period between sh14-11 and N14, these drastically changed DEGs may be related to the FW. After combining differential expression analysis across 11 pairs of tissues, fourteen genes were selected (Figure 6b). These genes display varied expression in flowers, fruit pulps, and fruit peels, potentially indicating fundamental disparities in parental germplasm. Importantly, these genes did not intersect with the identified QTL associated with FW.

**Figure 7:**
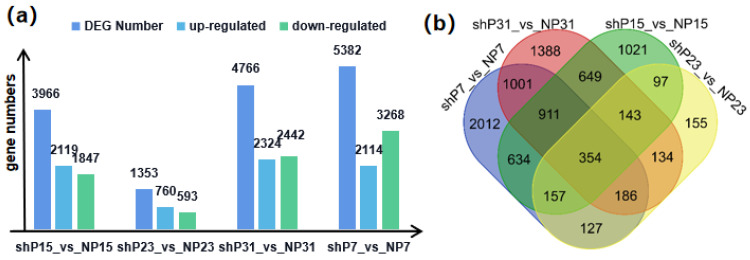
Identification of differentially expressed genes between sh14-11 and N14 in 4 pairs of tissues, including 4 stages of fruit peel (P7, P15, P23, P31). (**a**) The number and expression level changes of DEGs between sh14-11 and N14 in 4 pairs of tissues. (**b**) A total of 354 genes were differentially expressed across 4 pairs of tissues.

To unravel the genetic basis contributing to variations in FW in watermelon, we selected fruit peel for differential expression analysis between sh14-11 (shP7, shP15, shP23, and shP31) and N14 (nP7, nP15, nP23 and nP31). A total of 8969 DEGs were identified between sh14-11 and N14 (Figure 7a). Notably, 354 genes exhibited significant differential expression among the four stages (Figure 7b). Given the concurrent significant differences in FW at these stages, these 354 DEGs are potential key players influencing FW variations.

### 3.6. Co-Localization of QTL Mapping and Differentially Expressed Genes

In the findings, the genomic region spanning 31–38 Mb on chromosome 2 emerged as significantly associated with FW in the QTL mapping of 100 RIL materials. Simultaneously, BSA pinpointed the same region in the F_2_ population, characterized by distinct FW traits. The interval of 31–38 Mb was identified as a promising candidate region through both the ED and Δ(SNP-index) methods. Differential expression analysis between the two parental lines revealed 354 genes with potential relevance to FW. Consequently, co-locating these differentially expressed genes with the identified candidate intervals yielded 14 genes as notable candidates for FW variation (Supplementary Table S4).

**Figure 8:**
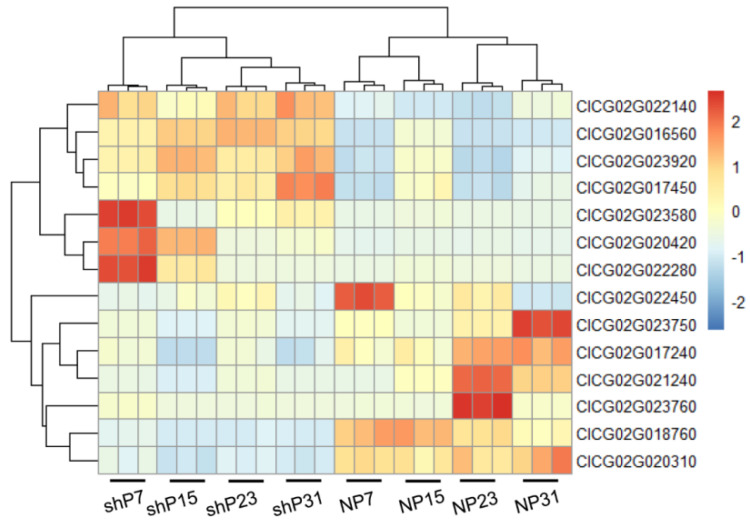
Changes in expression levels of 14 candidate genes in 4 pairs of tissues between sh14–11 (shP7, shP15, shP23, shP31) and N14 (NP7, NP15, NP23, NP31).

The expression patterns of these genes in the parents are different across the four stages of fruit peel (Figure 8). Among them, *ClCG02G022140*, *ClCG02G023920*, *ClCG02G016560*, *ClCG02G017450*, *ClCG02G023580*, *ClCG02G022280* and *ClCG02G020420* exhibit high expression in sh14-11, while *ClCG02G023750*, *ClCG02G022450*, *ClCG02G017240*, *ClCG02G018760*, *ClCG02G020310*, *ClCG02G021240* and *ClCG02G023760* show elevated expression in N14.

Utilizing the Non-Redundant Protein Sequence Database (NR), we conducted functional annotations for the 14 identified candidate genes. The majority of these genes are associated with pivotal roles in plant growth and development (Table 2). For instance, *ClCG02G022140* is annotated as a TGA transcription factor known to regulate the expression of target genes. Additionally, *ClCG02G022280* and *ClCG02G018760* are, respectively, identified as distinct constituent proteins of NAC transcription factors, which are recognized for their significant involvement in plant fruit development. Furthermore, *ClCG02G020420* is annotated as a zinc finger protein, and so forth. In summary, the differentially expressed genes we identified are likely to exert a substantial impact on the FW variation in watermelon.

## 4. Discussion

Up to now, there have been many studies on the construction of the watermelon genetic map, but most of them are based on low-density markers [4,5,31]. By deep sequencing the whole genome of 100 RIL lines, we constructed a high-quality genetic map containing 218,127 SNP markers and further completed the QTL mapping of FW. This method was successfully implemented across various species [32,33,34,35,36,37]. Furthermore, using the differential expression analysis of multiple tissues between parents to identify candidate genes saves a lot of time and energy compared to building a large population for fine positioning and speeds up the screening of candidate genes [38]. In the next step, rapid genotyping methods such as iBP-seq can be used for validating and verifying candidate genes [39], and the mutant phenotypes of the corresponding genes can be observed.

Previous studies on fruit size have mainly focused on FSI, FL and FD, with less emphasis on FW because the phenotype of FW is more affected by the environment and unstable. However, FW is a three-dimensional description of fruit size, which can also fully reflect the variation in fruit size. Some studies show that FW is a trait that is different from fruit size, but they are inherently correlated [22,40]. Previous reports demonstrated that the PVE for FSI, FL and FD could reach up to 31.9% [28]. In contrast, our QTL analysis for FW only accounted for 15.1% of the variation, suggesting a higher environmental influence on this trait. We identified the identical locus, located at 31–38 Mb on chromosome 2, using distinct methods in different populations—BSA in the F_2_ population and QTL mapping in RILs. This outcome underscores the mutual verification capability between the two mapping approaches, affirming the reliability of this locus. Additionally, the consistency in mapping results between the ED and Δ(SNP-index) indicates that various BSA methods exhibit concordant identification effects on loci with substantial impact [16].

In previous studies, many related loci and genes were identified for fruit size, such as *SUN* [41] and *lc* [42], proving their influence on fruit development in various species. The disparity between these known genes and our mapping interval might be attributed to variations in the genetic difference of lines. The two parents we selected both produce round fruits, leading to consistency in their FSI, making it challenging to pinpoint the locus related to FSI while focusing only on FW. Moreover, our lines differ from those used in prior FW mapping studies, potentially resulting in distinct QTLs and effect sites. Nevertheless, within our mapping interval, we identified not only homologous genes to known fruit size-related genes, such as *fas* [43], but also crucial transcription factors influencing fruit development, such as the TGA and NAC transcription factors.

*ClCG02G022140* was annotated as a TGA transcription factor in a previous study on Solanum lycopersicum. TGA transcription factors have been shown to influence fruit development. Specifically, *SlTGA2.3* interacts with the auxin amide hydrolase *SlILR5* to regulate cell division and participate in the fruit expansion process [44]. Additionally, RNA interference (RNAi) technology used to silence *SlTGA2.2* revealed changes in the early development and metabolism of Solanum lycopersicum fruits, leading to delayed fruit ripening [45]. *ClCG02G022280* and *ClCG02G018760* are annotated as NAC transcription factors, which play an important role in fruit ripening. In citrus, *CrNAC036* inhibits fruit ripening by synergistically down-regulating the expression of the ABA pathway gene *CrNCED5* through physical interactions with *CrMYB68* [46]. In kiwifruit, the AdNAC6 and AdNAC7 proteins function as transcriptional activators, binding to the promoters of AdACS1, AdACO1, AdMAN1 and AaTPS1, thereby activating their transcription and promoting fruit ripening [47]. These studies show that our mapping loci and candidate genes carry high credibility.

Overall, our findings successfully mapped FW trait to a hitherto undiscovered genomic region, employing a multifaceted approach, and identified candidate genes through differential expression analysis. This discovery holds promise for the development of molecular marker-assisted selection strategies in breeding watermelon fruits of varying sizes. Moreover, recognizing that FW can also be influenced by physiological and cultivation conditions, further investigation and functional verification are imperative to more precisely identify genes governing the watermelon FW. The cultivation of compact-sized watermelons is a pivotal aspect of watermelon variety development, characterized by aesthetically pleasant appearance, abbreviated growth cycles and enhanced portability. This investigation introduces valuable techniques and preliminary data for elucidating watermelon FW-related traits, expediting the watermelon breeding process and contributing significant to practical implications.

## 5. Conclusions

To dissect the genetic architecture of watermelon FW variation, we constructed 100 RIL lines from sh14-11 and N14 and sequenced the whole genome. A genetic map of 1649.42 cM was constructed, and 34–38 Mb on chromosome 2 was detected as a candidate interval for FW. Meanwhile, crossing sh14-11 and N14, a total of 100 plants in the F_2_ population were constructed, and the plants with extremely big fruit and small fruit were selected for BSA. Using Δ(SNP-index) and ED algorithms, a region spanning 31–38 Mb was detected on chromosome 2 that is related to FW, consistent with map-based mapping.

RNA-seq were carried out among 11 pairs of tissues between sh14-11 and N14. The expression profiles identified 1695 new genes and corrected the annotation of 2941 genes. After differential expression analysis in four stages between sh14-11 and N14, 354 differentially expressed genes were detected among four stages. Combining QTL mapping and differential expression analysis, 14 genes were located in the QTL mapping interval, including the annotated TGA and NAC transcription factors implicated in fruit development. We mined 14 candidate genes related to the FW by QTL mapping and differential expression analysis. It is believed that this identification is of great significance for genetic understanding and cultivation improvement in watermelon.

## Figures and Tables

**Table 1:** Distribution of single-nucleotide polymorphism markers on the high-density genetic map.

LG ID	Total Bin Marker	Total Distance (cM)	Average Distance (cM)	Max Gap (cM)	Gaps < 5 cM (%)
LG01	255	139.4	0.55	13.44	99.21%
LG02	234	164.39	0.71	11.95	99.57%
LG03	163	146.55	0.9	18.35	98.77%
LG04	211	154.58	0.74	8.92	96.19%
LG05	402	170.18	0.42	6.49	99.75%
LG06	236	148.78	0.63	14.5	98.72%
LG07	259	156.88	0.61	4.56	100%
LG08	135	123.92	0.92	11.95	99.25%
LG09	247	195.71	0.8	6.31	99.59%
LG10	280	146.09	0.52	11.95	98.92%
LG11	175	102.93	0.59	5.58	99.43%
Total	2597	1649.42	0.64	18.35	96.19%

**Table 2:** Location and annotation information for 14 candidate genes.

Gene ID	Chr	Start	End	Annotation
*ClCG02G022140*	2	36,608,687	36,616,809	transcription factor TGA9-like
*ClCG02G023920*	2	38,267,515	38,268,888	protein YLS3-like isoform X2
*ClCG02G016560*	2	31,036,756	31,040,967	protein ECERIFERUM 26-like
*ClCG02G017450*	2	31,950,483	31,952,384	probable protein phosphatase 2C 52
*ClCG02G023580*	2	37,923,184	37,929,184	linoleate 13S-lipoxygenase 2-1
*ClCG02G022280*	2	36,720,766	36,721,559	NAC domain-containing protein 90
*ClCG02G020420*	2	34,918,750	34,919,220	zinc finger protein ZAT12-like
*ClCG02G023750*	2	38,110,302	38,131,226	peroxidase 2-like
*ClCG02G022450*	2	36,884,417	36,885,709	protein FAF-like, chloroplastic
*ClCG02G017240*	2	31,779,639	31,782,992	probable E3 ubiquitin-protein ligase RHY1A
*ClCG02G018760*	2	33,491,641	33,496,029	NAC domain-containing protein 96
*ClCG02G020310*	2	34,854,388	34,855,109	Glyoxalase_2 domain-containing protein
*ClCG02G021240*	2	35,752,425	35,755,753	Lysine_decarbox domain-containing protein
*ClCG02G023760*	2	38,142,602	38,144,446	peroxidase 2-like

## Data Availability

All the original data in this study have been deposited into CNGB Sequence Archive (CNSA) of China National GeneBank DataBase. The bulked sergeant analysis datasets of fruit weight in the F_2_ population are deposited with accession number CNP0005458. The re-sequencing datasets of the RIL population are deposited with accession number CNP0005460, and the RNA-seq datasets of 22 samples are deposited with accession number CNP0005461.

## Supplementary Materials

The following supporting information can be downloaded at: https://www.mdpi.com/article/10.3390/genes15070933/s1. Supplementary Table S1: Data statistics for the resequencing of 100 RILs; Supplementary Table S2: Data statistics for the RNA-seq of 11 stages between sh14-11 and N14; Supplementary Table S3: Correlation coefficient of biological repetition for all samples; Supplementary Table S4: Expression levels of 14 differentially expressed genes in each sample.

## Abbreviations

AFLP	amplified fragment length polymorphisms
BSA	bulked segregant analysis
CIM	composite interval mapping
DEGs	differentially expressed genes
ED	Euclidean distance
FD	fruit width
FL	fruit length
FS	fruit shape
FSI	fruit shape indices
FW	fruit weight
LGs	linkage groups
NGS	next-generation sequencing
PCA	principal component analysis
PVE	phenotypic variation effect
QTL	quantitative trait loci
RILs	recombinant inbred lines
SNPs	single nucleotide polymorphisms
SSR	simple sequence repeat
WGR	whole-genome resequencing
